# Risk–Benefit Assessment of Consumption of Rice for Adult Men in China

**DOI:** 10.3389/fnut.2021.694370

**Published:** 2021-07-23

**Authors:** Haiqin Fang, Quantao Zhang, Shengjie Zhang, Tongwei Zhang, Feng Pan, Yufeng Cui, Sofie Theresa Thomsen, Lea S. Jakobsen, Aidong Liu, Sara M. Pires

**Affiliations:** ^1^China Center for Food Safety and Risk Assessment, Beijing, China; ^2^Yantai Huaxin Biomedical Science and Technology Co., Ltd, Yantai, China; ^3^School of Public Administration and Policy, Renmin University of China, Beijing, China; ^4^Liaoning Provincial Center for Disease Control and Prevention, Shenyang, China; ^5^Division of Diet, Disease Prevention and Toxicology, National Food Institute Technical University of Denmark, Lyngby, Denmark

**Keywords:** risk-benefit assessment, burden of disease, disability-adjusted life year, rice consumption, selenium, cadmium, inorganic arsenic

## Abstract

**Objective:** To evaluate the health impact of current and alternative patterns of rice consumption in Chinese adult men (40–79 years of age).

**Methods:** We applied a risk–benefit assessment (RBA) model that took into account the health effects of selenium (Se), cadmium (Cd), and inorganic arsenic (i-As). The health effects included the prevention of prostate cancer associated with exposure to Se, and an increased risk of lung, bladder, and skin cancer for i-As and chronic kidney disease (CKD) for Cd. We defined the baseline scenario (BS) as the current individual mean daily consumption of rice in the population of interest and two alternative scenarios (AS): AS1 = 50 g/day and AS2 = 200 g/day. We estimated the health impact for different age groups in terms of change in Disability-Adjusted Life Years (ΔDALY).

**Results:** The BS of rice consumption was 71.5–105.4 g/day in different age groups of adult men in China. We estimated that for AS1, the mean ΔDALY was −2.76 to 46.2/100,000 adult men of 40–79 years old. For AS2, the mean ΔDALY was 41.3 to 130.8/100,000 individuals in this population group.

**Conclusion:** Our results showed that, based on associated exposure to selenium, cadmium, and i-As in rice, the current consumption of rice does not pose a risk to adult men in China. Also, a lower (50 g/day) or higher (200 g/day) rice consumption will not bring larger beneficial effects.

## Introduction

Rice is the dominant staple food for over half of the population of the world, particularly in African and Asian developing countries (1). It is the second largest produced crop in the world, and China is the largest rice producer, with 27% of the global production in 2013 (2). In Asian developing countries, the consumption of rice contributes to around 70% of the daily energy intake from foods (3).

In China, almost 70% of the population chooses rice as a staple food. The “Chinese Dietary Guidelines 2016,” which are used as the cornerstone for nutrition guidelines of China, recommend a diet based on grains. Specifically, they recommend a daily individual intake of cereals of 250–400 g. The results of the “China Health and Nutrition Survey 1991 to 2011” (CHNS) showed that the consumption of rice was 200–300 g per day per person, but that there was a downward trend in the consumption of rice by adult residents in nine provinces in China. For example, the consumption of rice for urban residents has dropped from 257 g/day in 1991 to 177 g/day in 2011 (4, 5).

Rice is a source of nutrients, a complex matrix of proteins, carbohydrates, fats, fiber, many vitamins such as folic acid, thiamine, B vitamins, and trace minerals such as phosphorus, potassium, magnesium, and selenium (Se). In China, white rice, produced through a series of refining processes, is most commonly consumed. Unlike brown rice, which has outer bran and germ portions containing fiber, vitamins, and minerals, white rice loses a large amount of these components during refining processes (6–8). On the other hand, white rice is an important source of Se in China, and many prepackaged rice products in the market have claims on the label related to selenium content, such as “selenium-rich rice” (9). This has been motivated by low Se status in the population, which has been associated with an increased risk of overall mortality, poor immune function, and cognitive decline (10–13). On the contrary, the intake of Se has been associated with a decrease in the risk of several types of cancer, including prostate cancer (14).

However, in China, rice is also a source of exposure to toxic elements, such as cadmium (Cd), inorganic arsenic (i-As), and mercury (Hg) due to environmental contamination of fields and crops (15, 16). Exposure to these elements is associated with an increased risk of kidney disease, osteoporosis, cardiovascular disease (CVD), and cancer, respectively (15–21). Therefore, it is not straightforward to estimate whether changes in rice consumption in the Chinese population will be overall beneficial or detrimental to public health, and an integrated assessment of both risks and benefits is required.

For that purpose, risk–benefit assessment (RBA) of foods, a decision-support tool, is useful. An RBA weighs the risks and the benefits of food consumption against each other to support public health guidance and is particularly useful to develop or improve dietary guidelines for populations or population groups that may be at higher risk of diseases associated with the consumption of foods (22). In quantitative RBA, risks and benefits may be integrated into a composite health metric, usually the Disability-Adjusted Life Year (DALY). The DALY is also the preferred metric used for the estimates of the World Health Organization (WHO) of the global burden of foodborne diseases (23) and the Global Burden of Disease (GBD) Study (24). The difference in the sum of DALYs between a given reference scenario and one or more alternative scenarios (ASs) gives information on an overall health gain or loss by a theoretical intervention in a population, expressed in the loss of healthy life years.

In this study, we quantified the health impact of current and alternative patterns of rice intake in Chinese men using DALYs as a common health metric.

## Methods

### Identification of Components in Rice and Associated Health Effects

#### Selection of Components

We searched the literature for evidence on components in rice that may lead to beneficial and adverse health effects. Based on the strength of evidence on the relationship between exposure and potential health effects and on available data in China, we selected Se, Cd, and i-As and identified the potential health effects associated with exposure to these components through a narrative literature review. Hg was not included in this study, because it has been reported that the average intake of both total Hg (THg) and methylmercury (MeHg) for Chinese residents is far below the corresponding PTWI. Therefore, we assumed that THg intake and MeHg intake through rice for Chinese residents pose little risk (25).

Selenium, a naturally occurring non-metallic element, is an essential mineral required by the body in small amounts for normal physiological processes (10, 11). The human body obtains Se mainly through the diet, and the consumption of rice is one of the main sources of Se. In the past 20 years, the relevance of selenoproteins for health has been emphasized. Higher Se status (or Se supplementation) has antiviral effects, is essential for successful male and female reproduction, and reduces the risk of autoimmune thyroid disease (26). Among others, Se is associated with a decreased risk of different types of cancer. Prospective cohort studies have generally shown some benefits of higher Se status on the risk of prostate, lung, colorectal, and bladder cancers (15, 27, 28).

Cadmium (Cd), a heavy metal, is ubiquitous in the environment. Background soil concentrations are generally below 1 ppm but may range higher near metals mining and smelting operations, and due to the presence of Cd (up to 300 ppm) in phosphate fertilizer. Cd concentrations tend to be the highest in grains, nuts, legumes, and certain vegetables, but this is dependent upon Cd concentration in soil and agricultural practices (14, 16). There are reports in the literature that Cd is widely present in grains in China and that its content level is rice> wheat> potatoes> corn> miscellaneous grains (29). The exposure to Cd from rice accounted for ~56% of the total exposure to Cd (16). Cd absorption after intake is relatively low in humans (3–5%), but Cd is efficiently retained in the kidney and liver, with a very long biological half-life ranging from 10 to 30 years (30, 31).

Arsenic (As) is a metalloid naturally present in the crust of the earth and is widely distributed in the environment (soil, air, and water). It is well-known that the speciation of As plays an important role in determining As toxicity to humans. The inorganic species (i-As) is considered to be the most toxic form in As speciation (32). i-As is also the dominant form in Asian and European rice (32, 33). Between 30 and 100% of cereal crops are reported to be contaminated with i-As (33, 34). Exposure to i-As is associated with a wide range of adverse health effects, including neurotoxicity, diabetes, CVD, skin lesions, and various cancers (35).

#### Identification of Health Effects

We performed a narrative literature search to find relevant beneficial and adverse health effects of Se, Cd, and i-As observed in human and/or animal studies. We searched through Medline, google scholar, and China National Knowledge Infrastructure (CNKI). We used different combinations of more than 30 keywords, including “Selenium,” “Se, “human health,” “cancer,” “cardiovascular disease”, “diabetes,” “cadimum,” “Cd,” “Kidney”, “bone disease”, “ reproduction,” “inorganic arsenic,” “i-As,” “cancer,” “lung cancer,” “bladder cancer,” “skin cancer.” The search was limited to papers written in English or Chinese. We grouped studies according to the criteria for the strength of evidence proposed by the WHO (36). According to these criteria, the evidence can be graded as “convincing,” “probable,” “possible,” or “insufficient.” We only included evidence graded as “convincing” in this assessment.

Table 1 shows all identified health effects. For Se, eight beneficial health effects and one adverse health effect were identified. For Cd, five adverse health effects were identified. For i-As, three adverse health effects were identified. Of all these, the level of evidence was convincing for the decreased risk of prostate cancer, Keshan disease (KD), and Kashin–Beck symptoms for Se, and the increased risk, including selenium poisoning for Se and chronic kidney disease (CKD) for Cd, and lung cancer, bladder cancer, and skin cancer for i-As (Table 1).

**Table 1:** Description of the health effects associated with exposure to selenium (Se), cadmium (Cd), and inorganic arsenic (i-As), level of evidence, target population, and type of study.

**Nutrient/Chemical**	**Health effect**	**Level of evidence**	**Target population**	**Type of study**	**Health effects (beneficial/harmful/uncertain/no impact)**	**References**
Se	Type 2 diabetes	Conflicting	All population	Human studies Meta-analyses	Uncertain	(15, 37)
Se	Cardiovascular disease	Probable ramya Inconclusive	All population	Human studies ramya Meta-analyses	Beneficial	(12)
Se	Cancer (Prostate)	Convincing	Male	Human studies ramya Meta-analyses	Beneficial	WCRF
Se	Cancer (Bladder, lung)	Probable	All population	Human studies ramya Meta-analyses	Beneficial	(15, 38)
Se	Fertility and reproduction	Probable	All population	Human studies	Beneficial	(26)
Se	Seizures, Parkinson's disease, and cognitive decline	Probable mechanism unknown	Old people	Human studies	Beneficial	(10)
Se	Keshan disease	Convincing	All population	Human studies ramya Meta-analyses	Beneficial	(39)
Se	Kashin–beck symptoms	Convincing	All population	Human studies ramya Meta-analyses	Beneficial	(40)
Se	Selenium poisoning (Falling hair and nails)	Convincing	All population	Case reports	Adverse	(41)
Cd	Pneumonitis, destruction of mucous membranes	Probable mechanism unknown	Workers exposed to cadmium-containing fumes	Workers ramya Smokers	Adverse	(42)
Cd	Chronic kidney disease (CKD), Kidney Proteinuria, kidney stones, glomerular, and tubular damage	Convincing	All population	Human studies	Adverse	(17, 31, 43)
Cd	Reproductive System estrogen-like effects, affection of steroid-hormone synthesis	Probable mechanism known		Animal test	Adverse	(17)
Cd	Loss of bone density and mineralization, Itai-Itai disease, osteoporosis, and fracture	Probable	All population	Human study	Adverse	(42)
Cd	Carcinogenicity	Probable (only an uptake of cadmium *via* the respiratory system)		Animal test	Adverse	(17)
i-As	Lung cancer	Convincing	All population	Human studies	Adverse	[(17, 20, 35, 44) IARC, 2012, USFDA, 2016]
i-As	Bladder cancer	Convincing	All population	Human studies	Adverse	[(17, 21, 35, 44) IARC,2012, USFDA,2016]
i-As	Skin cancer	Convincing	All population	Human studies ramya Animal test	Adverse	[(17, 20, 35, 44) IARC,2012]

Recent epidemiological studies for prostate cancer showed that significant protective associations are consistently detected between Se and the risk of advanced, rather than localized or low-grade, prostate cancer (15, 27, 28, 45). KD is a Chinese endemic cardiomyopathy, initially established as of unknown cause (39). It was discovered in Keshan County, Heilongjiang Province, China, in the 1950s and the 1960s. When giving Se supplements to the residents of KD endemic areas, the incidence of KD was significantly reduced, and Se deficiency was established as a cause of KD (39, 46). Kashin–Beck disease (KBD) is a chronic endemic osteoarthropathy characterized by epiphyseal plate and deep cell necrosis of articular cartilage (40). It usually develops in childhood (5–13 years of age) and is mainly distributed in the oblique low-Se zone from northeast to southwest in China. A meta-analysis showed that all types of Se supplementation were of higher efficiency compared with the placebo in treating KBD in children (40). Moreover, an endemic disease (Se poisoning) was discovered in 1961 in parts of the population of Enshi County, Hubei Province of the People's Republic of China. During the years of the highest prevalence, from 1961 to 1964, the morbidity was almost 50% in the 248 inhabitants of the five most heavily affected villages; its cause was determined to be selenium intoxication (41). The most common sign of the poisoning was the loss of hair and nails. In areas of high incidence, lesions of the skin, nervous system, and possibly teeth may have been involved (41).

Long-term Cd exposure has been associated with kidney disease, osteoporosis, CVD, and cancer. Several studies, including biomarkers monitoring, case–control studies, cohort studies, and meta-analyses, have reported that long-term dietary exposure to Cd can cause damage to the kidney and bone (30, 31, 42, 47).

Humans are mainly exposed to i-As through drinking water and foods (48). Consistent evidence of dose–response relationships has been proven for lung, bladder, and skin cancer, primarily based on observational epidemiological studies in regions where geologic i-As is endemic and chronic exposure is high (26, 48, 49).

Based on the collected evidence, we selected the benefit of prevention of prostate cancer given exposure levels to Se, the risk of CKD with different exposures to Cd, and the risk of lung, bladder, and skin cancer with different exposures to i-As to be evaluated in the model. We focused our analysis on adult men older than 40 years because the evidence of the beneficial effects of Se was based on this population group. Moreover, epidemiological data for Cd were based on individuals >40 years (50).

### Baseline Scenario (BS) and AS

We defined as BS the current average individual daily consumption of rice in China in different male population groups (**Table 3**; from 71.5 to 105.4 g/day, depending on population group) and two alternative consumption scenarios (AS). The recommended daily intake of staple foods (including grains, beans, and potatoes) defined in the “Chinese Dietary Guidelines 2017” is 250–400 g/day, including 50~150 g of whole grains and miscellaneous beans and 50~100 g of potatoes (51). To evaluate the health impact of changing the consumption to both lower and higher levels than the current, the AS were defined as follows: AS1: 50 g/day; AS2: 200 g/day.

### Exposure Assessment

Data on the rice consumption in the Chinese population were collected from the National Food Consumption Survey, which was conducted in 2017–2018 and interviewed 25,812 participants (data not published). The survey collected food consumption data with a 24-h dietary recall method on three consecutive days. It is representative of the Chinese population in terms of gender, age distribution, and geography.

Data on the concentration of Se and Cd in rice were collected from a total of 19,786 individual rice samples collected from supermarkets, local markets, and in the field during harvest time in 31 provinces, autonomous regions, and municipalities of China from 2011 to 2015 (16). Se and Cd were detected by inductively coupled plasma atom–mass spectrometry (ICP–MS) following the protocol for elemental analysis in the China National Monitoring Handbook of Food Safety and China National Food Safety Standard GB/T 5009.268-2016 (52). The concentration of i-As in rice was collected from the literature reported by Qian et al. (14).

For each population group and scenario, exposure to Se, Cd, and i-As from rice was calculated by the equation below.

(1) $$\begin{array}{r} Exposure\;\left(\mu g\;/d\right)=rice\;consumption\;\left(g/d\right)\times \\ \;Se\left(Cd\right)\left(i-As\right)\;concentration\;in\;rice\;\left(\mu g\;/g\right) \end{array}$$

### Benefit and Risk Characterization

We characterized the attributable risk of prostate cancer given exposure levels to Se; the attributable risk of CKD associated with different exposures to Cd; and the attributable risk of lung cancer, bladder cancer, and skin cancer associated with different exposures to i-As in each of the scenarios (BS and AS).

#### Selenium Model

The dose–response relationship between Se and prostate cancer was obtained from a meta-analysis conducted by the World Cancer Research Fund (WCRF) report (45), assuming a relative risk (RR) of 1 at zero consumption and a log-linear association (52):

(2) $$\begin{array}{r} \text{ln}\left(\text{RR}\right)=\beta \text{x} \end{array}$$

where *x* is the intake amount and β can be estimated from the RR for a given *x*.

The summary RR per 10 μg/L in plasma or serum was 0.95 (95% CI 0.91–1.00) (45).

According to the logarithmic regression equation for dietary selenium intake in China based on cereals, 10.0 μg/L in plasma or serum is equal to 1.4 μg/d selenium intake from food (53). According to Equations (2) and (3), β is calculated as −0.0366, and RR for the reference (RR_r_) and each AS (RR_a_) is calculated as:

(3) $$\begin{array}{r} RR=e^{\wedge \beta dose}={\text{e}}^{\wedge -0.036638dose} \end{array}$$

(4) $$\begin{array}{r} \text{RRr}={\text{e}}^{\beta dose\left(refer\right)}\text{orRRa}={\text{e}}^{\beta dose\left(alternative\right)} \end{array}$$

Based on the data from the GBD 2013 for China (54), the health impact of prevention of prostate cancer attributable to Se intake was estimated by calculating the change in DALY using the so-called potential impact fraction (PIF) (52, 55). The PIF compares the RR estimates associated with the intake in each scenario, where RR_r_ is the RR at exposure level in the reference scenario and RR_a_ is the RR at exposure level in the AS. It reflects the proportion of the disease burden attributable to the change in intake of a food or a food component. It is estimated as:

(5) $$\begin{array}{r} PIF=\frac{RR\text{r}-RRa}{\text{RRr}} \end{array}$$

The net health effect for Se in the two ASs (ΔDALY) was calculated using the following formula:

(6) $$\begin{array}{r} \Delta DALY=DALY_{\;Alternative\;Scenario}-DALY_{Reference\;Scenario}\\ =\;PIF^{*}DALY=-{\left[\frac{RRr-RRa}{RRr}\right]}^{*}DALY \end{array}$$

#### Cadmium Model

To estimate the health impact of exposure to Cd through different consumption scenarios, we adapted the model developed by Zang et al. (56). The increase in the CKD prevalence due to Cd exposure was simulated based on a previously reported pharmacokinetic model describing the relationship between dietary cadmium intake and urinary cadmium (UCd), as well as a previously published dose–response relationship between UCd and the glomerular filtration rate (GFR) (57).

##### Deriving CKD prevalence using GFR

Chronic kidney disease is categorized in five stages that are mainly based on the GFR, according to the National Kidney Foundation guideline (58). The early stages (stages 1–3) of CKD usually do not show clinical symptoms and are not considered in the assessment. However, the late-stage CKD (stage 4–5), characterized by severe decreases in the GFR, requires clinical interventions such as dialysis or a kidney transplant. In this assessment, stage 4 and stage 5 CKD are defined according to the National Kidney Foundation's guideline as conditions with GFR 15–30 ml/min/1.73 m^2^ and <15 ml/min/1.73 m^2^, respectively. Since GFR generally follows a normal distribution in the general human population, the prevalence of stage 4 and stage 5 CKD can be modeled using the cumulative density function from a normal distribution given the mean and the SD.

##### Deriving Age-specific CKD prevalence

The Cd-attributable annual probability of being diagnosed with stage 4 and stage 5 CKD was then estimated as the difference between age-dependent but Cd-independent risk of stage 4 and stage 5 CKD and the Cd- and age-dependent risk of stage 4 and stage 5 CKD as described in the study by Zang et al. (56). The probability for individuals >40 years of age was calculated as the difference between the probability at the individual current age and that at the individual current age +1 year, both with and without Cd exposure as described in the study by Herrera et al. (50).

In general, after age 30–40, GFR declines by about 0.8 ml/min/1.73 m^2^ per year in healthy populations. Assuming that this rate of decline is consistent through life after 40 years old, and using a published mean GFR (x_α_) obtained for a population with an average age of α, the mean GFR for an older population of the same geographic area (x_α+*n*_) can be modeled as:

(7) Xa + n,cd = (Xa−0.8n)(1−0.078(Ucd−1.0)

where *n* is the number of years the calculated population is older than the published population.

For stage 4 CKD, we assumed no excess mortality. Therefore, stage 4 CKD DALYs were given by the Years Lived with Disability (YLD) component, obtained by multiplying the incidence rates with the stage 4 CKD duration and disability weight (59). We applied a lifelong duration, corresponding to the age group-specific national life expectancy, which was derived from the 2015 revision of the National Population Data (available at: https://data.stats.gov.cn). Therefore, the equation for calculating DALY at stage 4 CKD is:

(8) $$\begin{array}{r} \text{DALY}\left(stage4\right)=\text{YLD}\left(stage4\right)=attributable\;disease\\ \;incidence\left(stage4\right)\times disability\;weight\times \\ \;\left(national\;life\;expectancy-age\;of\;onset\right) \end{array}$$

For stage 5 CKD (end-stage renal disease), we assumed a 100% case fatality ratio as described in the study by Zang et al. (56). Thus, YLD for this stage was defined as 0. Years of Life Lost due to premature death (YLLs) were calculated by multiplying the number of deaths with the age group-specific residual life expectancy. In accordance with the WHO Global Burden of Foodborne Disease and WHO Global Health Estimates, we used the highest projected life expectancy for 2050 by Standard Expected Years of Life Lost (SEYLL) for 2050 (60) as the normative life expectancy table. The equation for calculating DALY at stage 5 CKD is:

(9) $$\begin{array}{r} \text{DALY}\left(stage5\right)=\text{YLL}\left(stage5\right)=attributable\;disease\\ \;incidence\left(stage5\right)\times \text{SEYLL} \end{array}$$

#### i-As Model

To estimate the health impact of exposure to i-As through different consumption scenarios, we adapted the model developed by Jakobsen et al. (35). The used dose–response relationships are shown in Table 2.

**Table 2:** Dose–response relationships of the risk of lung, bladder, and skin cancer as a function of dietary exposure to inorganic arsenic (i-As)^a^.

**Cancer type**	**Extra lifetime risk of cancer^b^**
Lung	*r* = 10^−5^x^2^+0.001x
Bladder	*r* = 10^−6^x^2^+0.0004x
Skin	*r* = 0.0015x

a *Dose–response relationships were established by US FDA and Integrated Risk Information System (35)*.

b *r is extra lifetime risk of cancer, as a function of the exposure; x is equal to the lifetime daily dose of i-As*.

The annual incidence (AI_c_) of lung, bladder, and skin cancer (c) for each sex (s) caused by dietary exposure to i-As was estimated by:

(10) $$\begin{array}{r} {\text{AI}}_{\text{c}}=\left(\text{Npop},\text{s}\times {\text{r}}_{\text{c}}\right)/\text{LE} \end{array}$$

where Npop,s is the size of the exposed population per sex, r is the extra lifetime risk per sex of each cancer, c, and LE is the longest projected life expectancy for 2050 (60).

To estimate DALYs, we applied an incidence-based approach and used the mean DALYs for lung cancer, bladder cancer, and skin cancer (44). The disability weights for lung cancer, bladder cancer, and skin cancer were collected from the literature and set to 0.15, 0.09, and 0.05, respectively (61). The mean DALYs of each case of lung cancer, bladder cancer, and skin cancer were calculated using the DisMod II software and WHO disease Burden Excel template (62), combined with the number of cases of three cancers in China in 2013 (63):

(11) $$\begin{array}{r} {\text{DALY}}_{\text{iAs}}={\text{AI}}_{\text{c}}\times {\text{DALY}}_{\text{ave}/\text{case}} \end{array}$$

All calculations were done in SPSS 17.0 statistical software.

## Results

### Exposure to Cd and Se Through Rice Consumption

The concentration of Se, Cd, and i-As in rice was 0.085, 0.062 ± 0.128 (16), and 0.119 ± 0.079 μg /g (14), respectively. The actual rice consumption in the different age groups (BS) and the estimated exposures to Se and Cd for the BS and AS are shown in Table 3.

**Table 3:** Exposure assessment of Se and Cd for baseline and alternative scenarios.

**Age group**	**40–49**	**50–54**	**55–59**	**60–64**	**65–69**	**70–74**	**75–79**	**AS1**	**AS2**
Rice consumption (g)	101.5	106.7	97.8	97.0	91.5	89.6	83.7	50	200
Se intake (μg)	8.63	9.07	8.31	8.24	7.77	7.61	7.11	4.25	17.0
Cd exposure (μg)	6.29	6.62	6.06	6.01	5.67	5.55	5.19	3.1	12.4
i-As exposure (μg)	20.13	21.16	19.40	19.24	18.15	17.77	16.60	9.92	39.67

### Beneficial Health Impact of Selenium

Table 4 shows the estimated net health impacts for Se of the two scenarios (DALY). Results showed that AS2 would lead to a positive health impact when comparing with the BS in all age groups (i.e., negative ΔDALY), while AS1 would lead to a negative health impact. The magnitude of these impacts was larger in older age groups.

**Table 4:** Dose–response parameters and estimated net health impact (ΔDALY) associated with intake of Se for the prevention of prostate cancer through the consumption of rice in alternative scenarios 1 and 2 in different age groups.

**Age group (years)**	**40–49**	**50–54**	**55–59**	**60–64**	**65–69**	**70–74**	**75–79**	**AS1**	**AS2**
Beta dose	−0.3162	−0.3323	−0.3045	−0.3019	−0.2847	−0.29897	−0.2605	−0.1557	−0.6228
RR	0.7289	0.7173	0.7375	0.7394	0.7523	0.74159	0.7707	0.8558	0.5364
PIF AS1	0.1741	0.1932	0.1604	0.1574	0.13765	0.15402	0.1105		
ΔDALY for AS1 (per 100,000 adult men)	0.54	0.33	0.92	5.08	8.87	26.83	48.03		
PIF AS2	−0.2641	−0.2521	−0.2727	−0.2745	−0.2869	−0.2767	−0.3040		
ΔDALY for AS2 (*per* 100, 000 *adult men*)	−0.73	−6.37	−13.11	−26.94	−55.56	−110.05	−164.75		

### Adverse Health Impact of Cd

Table 5 shows the estimated net health impacts for Cd for the two scenarios. Results showed that AS2 would lead to a negative health impact comparing with the BS. We could not observe the Cd impact of AS1, because the UCd at 1.0 μg/g creatinine was used as the threshold for an adverse outcome estimation, and all estimated UCds in AS1 were below 1.0.

**Table 5:** Estimated cadmium–related GFR and net health impact (ΔDALY) associated with CDK4^*^ and CDK5^**^ through the consumption of rice in alternative scenario 2 in different age groups.

**Age group (years)**	**40–49**	**50–54**	**55–59**	**60–64**	**65–69**	**70–74**	**75–79**
Middle age	45	52	57	62	67	72	77
GFR for RS (Age, –Cd)	104.3	99.3	95.3	91.3	87.3	83.3	79.3
GFR for AS2^*^ (Age, +Cd)	104.3	98.1	93.8	89.6	85.4	81.2	77.2
SEYLL (61)	59.13	40.41	35.55	30.73	25.96	21.31	16.89
ΔDALY for CDK4 in AS2	32.32	7.98	9.98	10.02	7.46	2.22	−5.07
ΔDALY for CDK5 in AS2	56.44	62.14	99.13	138.09	173.10	197.30	204.79

^†^*GFR, Glomerular filtration rate*.

* *CDK4, Chronic kidney disease, stage 4*.

** *CDK5, Chronic kidney disease, stage 5*.

### Adverse Health Impact of i-As

We estimated an increased positive health impact associated with lung cancer, bladder cancer, and skin cancer due to exposure to i-As in AS1 in adult men older than 40 years, and an increased negative health impact associated with the three cancers due to exposure to i-As in these adult men in AS2 (Table 6).

**Table 6:** Estimated net health impact (ΔDALY) associated with the intake of inorganic arsenic through the consumption of rice in alternative scenarios 1 and 2 in different age groups.

**Age group (years)**	**40–49**	**50–54**	**55–59**	**60–64**	**65–69**	**70–74**	**75–79**
**Lung cancer**							
Incidence of RS	3551.32	392.51	322.61	217.63	136.82	113.24	75.47
Incidence of AS1	162.42	17.07	15.31	10.42	6.94	5.87	4.19
Incidence of AS2	651.61	68.50	61.44	41.79	27.85	23.54	16.80
ΔDALY for lung cancer in AS1 (per 100,000 adult men)	−1.52	−1.68	−1.41	−1.39	−1.23	−1.17	−1.00
ΔDALY for lung cancer in AS2 (per 100,000 adult men)	2.92	2.77	3.03	3.05	3.22	3.27	3.45
**Bladder cancer**							
Incidence of RS	131.82	14.57	11.98	8.08	5.08	4.20	2.80
Incidence of AS1	64.92	6.82	6.12	4.16	2.77	2.35	1.67
Incidence of AS2	259.87	27.32	24.50	16.67	11.11	9.39	6.70
ΔDALY for bladder cancer in AS1 (per 100,000 adult men)	−0.27	−0.29	−0.25	−0.24	−0.21	−0.20	−0.17
ΔDALY for bladder cancer in AS2 (per 100,000 adult men)	0.51	0.48	0.53	0.53	0.56	0.57	0.60
**Skin cancer**							
Incidence of RS	494.08	54.60	44.89	30.28	19.04	15.76	10.50
Incidence of AS1	243.39	25.59	22.95	15.61	10.40	8.79	6.27
Incidence of AS2	973.55	102.35	91.79	62.43	41.61	35.18	25.10
ΔDALY for skin cancer in AS1	−1.02	−1.13	−0.95	−0.93	−0.82	−0.79	−0.67
ΔDALY for skin cancer in AS2	1.96	1.85	2.03	2.04	2.15	2.19	2.30

### Risk–Benefit Assessment

**Figure 1:**
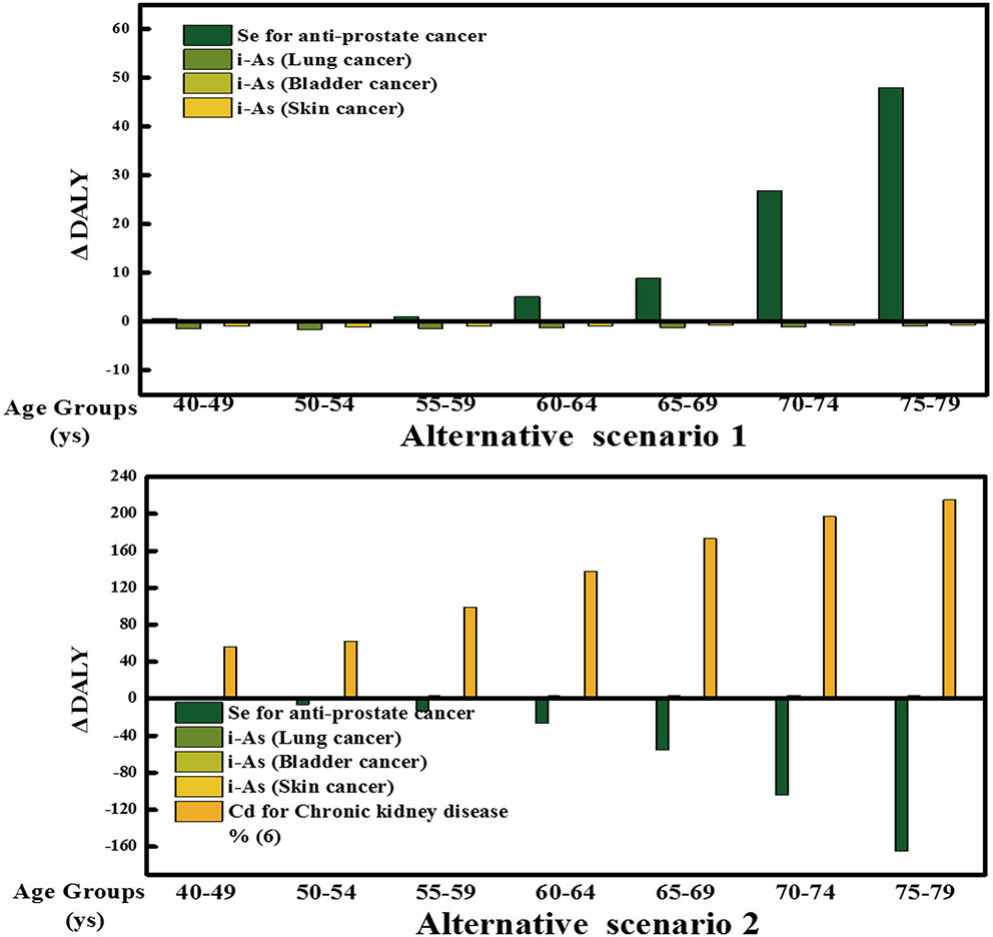
Difference in DALYs by scenario and outcome. Contribution of each health outcome to the overall DALY difference estimates for each AS for the Chinese adult men (40–49, 50–54, 55–59, 60–64, 65–69, 70–74, 75–79 years old). Each bar represents the health impact of the Se, Cd, and i-As on individual health effects.

**Figure 2:**
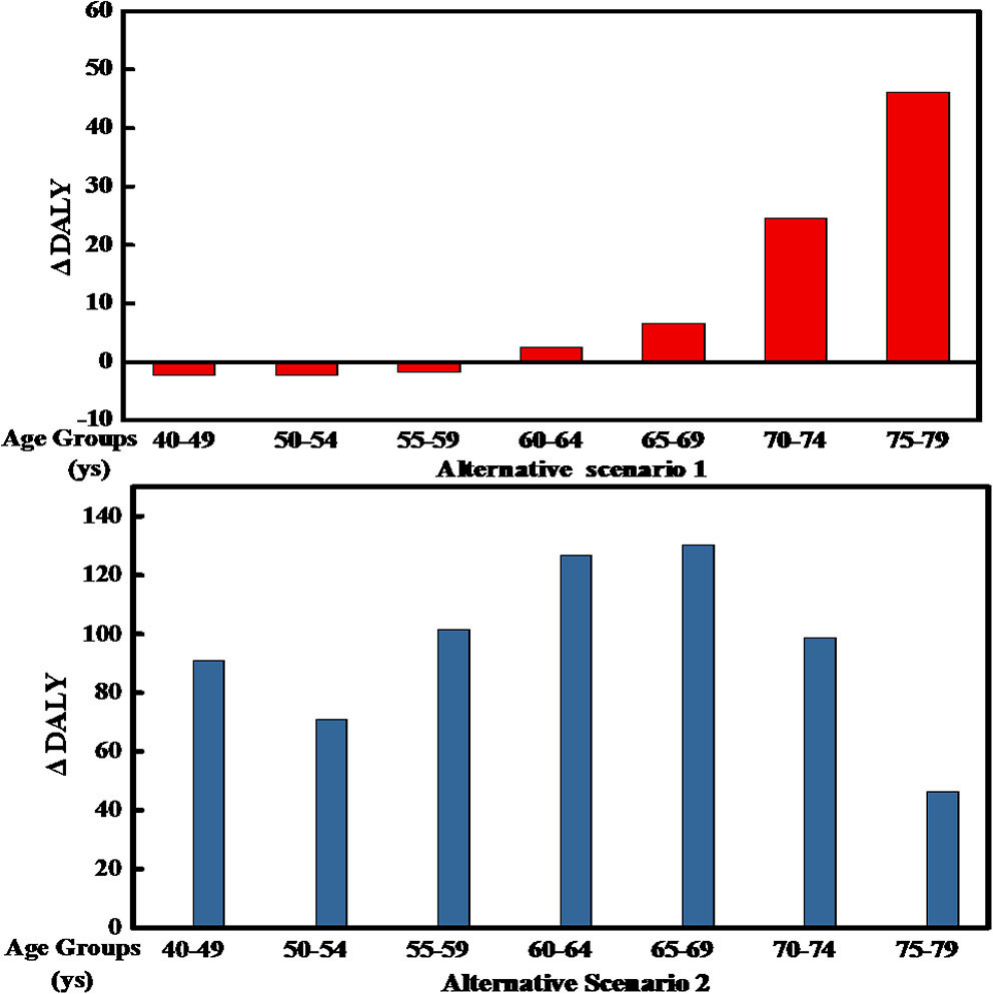
Difference in Disability-Adjusted Life Years (DALYs) for different scenarios and age groups of Chinese men. The bars represent the DALY difference between each group in the two ASs and the current consumption.

The estimated overall health impact and outcome of the Se, Cd, and i-As of rice consumption in each scenario and age group (from 40–49 to 75–79 years old) is shown in Figure 1. Table 7, Figure 2 show the RBA result of Se, Cd, and i-As for each AS compared with the BS.

**Table 7:** Risk–benefit assessment of two alternative rice consumption scenarios compared to the baseline consumption in different age groups of adult men in China (in Disability–Adjusted Life Year difference, ΔDALY).

**Age group**	**40–49**	**50–54**	**55–59**	**60–64**	**65–69**	**70–74**	**75–79**
ΔDALY in AS1*	−2.27	−2.76	−1.69	2.51	6.61	24.67	46.20
ΔDALY in AS2*	93.42	68.85	101.59	126.78	130.83	95.28	41.33

*AS, Alternative scenario*.

*The assessment takes into account the health effects associated with exposure to Se, Cd, and i–As*.

For AS1, we estimated a health loss for the age group of 60–79-year adult men, but a health gain for the population between 40 and 59 years. The opposite was observed for AS2: We estimated a health loss for men in the age group of 40–79 and a health.

## Discussion

In this study, we estimated the risk–benefit balance of different scenarios of rice consumption in men > 40 years of age in China. We assessed the associated health effects of Se in preventing prostate cancer, adverse effects of Cd-induced CKD, and effects of As-induced lung, bladder, and skin cancer. Our results showed that the current consumption of rice (83.7–106.7 g/day) in this population group does not lead to an adverse health impact. They also showed a negative health effect (i.e., a loss of life years ranging from 2.5 to 46.2 per 100,000) if men aged between 60 and 79 consume a lower amount of rice (50 g/d), and a loss of life years ranging from 41.3 to 130.8 per 100,000 when adult men (40–79) consume higher amounts of rice (200 g/d). There are two important factors influencing our estimates. One is the incidence and mortality of prostate cancer significant increase in China in recent years (54); the other is that the incidence of prostate cancer and CKD increases rapidly with age.

Rice is the main source of carbohydrates in many Asian populations, including the Chinese. Rice consumption has dropped significantly in recent years, from ~200 g per person per day in 2011 (4, 5) to 100 g in 2018. The structure of the traditional agricultural industry of China and the dietary habits of the population are undergoing changes. Consumer demand is affected by these changes, as showed by the increased consumption of pork, chicken, eggs, milk, and soy, and the decreased rice consumption rice (4, 64). However, the dietary guidelines of China recommend an intake of grains 250–400 g/day, including rice and whole grains.

Assessing the health impact of the exposure to nutrients and potentially toxic elements through its consumption in rice is relevant due to its importance as a staple food in the country. White rice in China is an important source of Se, absorbed, and accumulated by plants (65). Many epidemiological studies (cohort studies and randomized control trials) and meta-analyses have shown that there is an inverse relationship between serum Se levels and prostate cancer risk (28). The adult intake of Se recommended by the Chinese Nutrition Society is 50 μg/d (66), but varies with age group, pregnancy, and breastfeeding.

Cd is a well-known metal imposing threats to human health, and it is easily absorbed by plants and accumulated in plants. Studies have found that different crops have different levels of cadmium absorption. The enrichment coefficient is rice> soybeans> barley> corn> wheat (29). Cd can accumulate in polished rice over the permitted range of 0.2 mg kg according to Chinese standard (GB 2762-2017) (67).

Arsenic is a naturally occurring metalloid in soil, air, water, and food in organic and inorganic forms. Several epidemiological studies have shown that inorganic i-As is carcinogenic to humans. It has been reported that 25% of milled rice samples in China contained i-As in excess of the maximum allowable concentrations (MAC, 0.15 mg/kg) established by Chinese legislation (14, 67).

In our study, we strictly graded the level of evidence of the associations between the exposure to a component in food and the associated health effects. We based this on the WHO criteria, the criteria set by the WCRF, and the risk–outcome pairs in the GBD study (36). According to these criteria, many beneficial health effects of Se, such as KD (39, 46), KBD (40), protecting cancer or cardiovascular (68), were not included in our model due to the lack of a clear dose–response relationship. Had the evidence been strong and these health outcomes been included in our study, the overall estimated health impact would have changed.

Our substitution model was based on deterministic approaches, assuming that all individuals would substitute in the same manner. Thus, our model did not take variability in the substitution into account, apart from baseline consumption in different age groups. The data, assumptions, and models applied in this RBA all contribute to the uncertainty in the overall health impact of the substitutions we investigated. We were able to quantify some but not all of these uncertainties. Table 8 lists the sources of unquantified uncertainty in our study and explains the potential impact on the final results. We generally applied a conservative approach and overestimated especially toxicological risks. Still, the impact and direction of other sources of uncertainties are difficult to characterize.

**Table 8:** Unquantified sources of uncertainty of the final DALY difference estimates.

	**Source of uncertainty**	**Impact**
Health outcome	Identification of relevant nutrients and compounds	There may be other compounds with adverse/ beneficial effects present in rice that have not been accounted for in this RBA.
	Identification of relevant health effects	There may be other health effects associated with the consumption of rice which was not included in this RBA
	Identification of relevant subgroups	There may be other relevant subgroups in relation to the health effects considered in this RBA
Exposure assessment	Uncertainty in consumption data	Over– or under-estimation of consumption. We did not adjust for within-individual variability in consumption, which may cause the overestimation of upper and lower tails of distributions of consumption amounts
	Uncertainty in concentration data	There may be large uncertainty associated with measuring nutrient and contaminant concentrations in food.
Health impact characterization	Choice of dose–response modeling	Uncertainty is associated with the fitted dose–response model to describe Cd and i-As-induced health effects, which may lead to uncertainty around the dose estimated to cause an adverse effect in rats. We most likely overestimated the risks
	Choice of critical effect size for Se-, Cd-, and i-As-induced health effects	Large uncertainty is associated with establishing critical effect size used for Se, Cd, and i-As dose–response modeling, leading to additional uncertainty around the critical effect dose for Se-induced health effects.
	RR estimates based on epidemiological observational studies	The RR estimates describing the association between food consumption and disease, derived from observational studies, may already be based on underlying food substitutions. This causes uncertainty around the overall health impact of the substitution.
	Dose–response models based on epidemiological data	Large uncertainty is associated with the assumption on linearity of the RR dose–response relations applied. We most likely underestimated the benefits associated with the substitution
DALY estimation	Choice of distributions to describe uncertainty around DWs	Uncertainty is associated with the assumptions on the PERT distribution being suitable to describe the uncertainty around the DWs
	Choice of onset and duration of disease	Large uncertainty associated with the assumptions on onset and duration of disease, which may lead to either over- or under-estimation of the final DALY estimates. Likewise, we assumed no time-lag from exposure to disease, which is also associated with great uncertainty. In contrast to all other health effects considered, for the Cd and i-As-induced health effects we applied lifetime probabilities and not annual probabilities of disease, causing an overestimation of the risks associated with Cd and i-As exposure.
Overall evaluation of unquantified uncertainty		In general, we applied a conservative approach when making assumptions favoring especially toxicological risks associated with the consumption of rice. However, uncertainties around unidentified compounds or health effects may as well cause an underestimation of risks.

*Bw, body weight; DALY, Disability–Adjusted Life Year; DW, disability weight; RBA, risk–benefit assessment; RR, relative risk*.

Although there have been many reports associating rice as a staple food with the risk of type 2 diabetes (6), we did not include it in our study. Our study focused on the analysis of the risks increased by the adverse effects of pollutants, such as Cd, i- As, and benefits with the health effects of nutrients, such as Se, in rice. To perform an assessment of Type 2 diabetes risk and consumption rice, a comparison with consumption of whole grain would be needed (8).

We also did not include other important nutrients or chemicals contained in rice, such as vitamin B family, dietary fiber, or mercury. Previous studies have reported that both exposure to heavy metals through foods and insufficient cereal food consumption cause a high disease burden globally. According to Oberoi et al., the global annual DALYs caused by arsenic-driven cancer and CHD is 1.4 and 49 million, respectively (69). Cd has been estimated to result in 70,513 DALYs and 2,064 deaths globally (56). The GBD study showed that the burden from diseases attributable to six dietary factors, at the global scale, including low in whole grains, accounted for more than 1% of global DALYs (70). These estimates demonstrate the need for encouraging consumers to increase cereal consumption, while monitoring and reducing contamination of foods with heavy metals. Low intake of whole grain foods led to 4 million DALYs and 250,000 CVD deaths.

It should be mentioned that human diets are complex, and several variables and determinants related to the food exposure of Se, Cd, and i-As were not considered in our work. For example, although rice is a staple food in China, other cereals such as wheat and corn also play an important role in the Chinese daily diet, and these cereals also contain Se, Cd, and i-As composition. Specifically, the concentration of Se, Cd, and i-As concentration in Chinese wheat was 0.0742 ± 0.0211 μg/g (71), 0.0069–0.0085 μg/g (72), and 0.152 μg/g (73), respectively. It was reported that corn contains 0.05–14.5 μg/g Se (74), 0.01–0.54 μg/g Cd (43), and 0.125–0.286 μg/g total As (75). These data indicate that the risk and benefit outcomes studied in our model should be complemented taking into account the intake of other foods in the Chinese population.

Furthermore, we did not conduct our assessment for different regions in the country. China is a large country, and there are differences in Se concentration between different parts of the country, suggesting the possibility of toxicity caused by higher and lower amounts of Se consumption (76). Besides, as harmful elements are related to industrialization and urbanization, the soil concentration of Cd and i-As also varies between different parts of China (33, 77). Due to the accumulation effect, elements in soil could contribute to the distribution of elements in grain. Evaluating such differences would require data on the origin of rice and its implication on the concentration of food components in rice.

Risk–benefit assessment is increasingly used to inform dietary advice and other public health strategies for diet-associated disease prevention (78, 79). To our knowledge, this is the first RBA that quantifies the health impact of Se, Cd, and i-As of rice in terms of DALYs. By quantifying the health impact of adherence to dietary guidelines, our study provides data basis for national public health policy, such as the revision of dietary guidelines of China and setting the limitation standard of cadmium or i-As content in rice.

## Data Availability Statement

The raw data supporting the conclusions of this article will be made available by the authors, without undue reservation.

## Author Contributions

HF: design, model building, statistics, and drafting. QZ: literature review, drafting, and editing. SZ: model building. TZ: literature review and health effect assessment. FP and YC: consumption calculation. ST and LJ: checking about methods and editing. AL and SP: correspondence, design, and checking about methods and results. All authors contributed to the article and approved the submitted version.

## Conflict of Interest

QZ was employed by the company Yantai Huaxin Biomedical Co., Ltd. The remaining authors declare that the research was conducted in the absence of any commercial or financial relationships that could be construed as a potential conflict of interest.

## Publisher's Note

All claims expressed in this article are solely those of the authors and do not necessarily represent those of their affiliated organizations, or those of the publisher, the editors and the reviewers. Any product that may be evaluated in this article, or claim that may be made by its manufacturer, is not guaranteed or endorsed by the publisher.
